# The sentinel node approach in gynaecological malignancies

**DOI:** 10.1007/s40336-016-0187-6

**Published:** 2016-06-13

**Authors:** Angela Collarino, Sergi Vidal-Sicart, Germano Perotti, Renato A. Valdés Olmos

**Affiliations:** 1 Institute of Nuclear Medicine, Università Cattolica del Sacro Cuore, Largo F. Vito, 1, 00168 Rome, Italy; 2 Nuclear Medicine Section, Department of Radiology, Leiden University Medical Center, Albinusdreef 2, 2333 ZA Leiden, The Netherlands; 3 Department of Nuclear Medicine, University Hospital Clínic Barcelona, Villarroel, 170, 08036 Barcelona, Spain; 4 Interventional Molecular Imaging Laboratory, Department of Radiology, Leiden University Medical Center, Albinusdreef 2, 2333 ZA Leiden, The Netherlands; 5 Department of Nuclear Medicine, The Netherlands Cancer Institute-Antoni van Leeuwenhoek Hospital, Plesmanlaan 121, 1066 CX Amsterdam, The Netherlands

**Keywords:** Cervical cancer, Endometrial cancer, Vulvar cancer, Sentinel lymph node, SPECT/CT

## Abstract

This review discusses the state-of-the-art of sentinel lymph node mapping in gynaecological malignancies, including cervical cancer, endometrial cancer, and vulvar cancer, with an emphasis on new technological advances. For this objective, PubMed/MEDLINE was searched for relevant studies about the sentinel lymph node procedure in gynaecology. In particular, the use of preoperative lymphatic mapping with lymphoscintigraphy and single photon emission tomography/computed tomography (SPECT/CT) was identified in 18 studies. Other recent advances as hybrid tracers (e.g. ICG-^99m^Tc-nanocolloid) and intraoperative tools (portable γ-camera and 3D navigation devices) appear to also represent a useful guide for the surgeon during the operation. Concerning vulvar and cervical cancers, the sentinel lymph node procedure has been incorporated to the current guidelines in Europe and North America, whereas for endometrial cancer it is considered investigative.

## General introduction

**Fig. 1:**
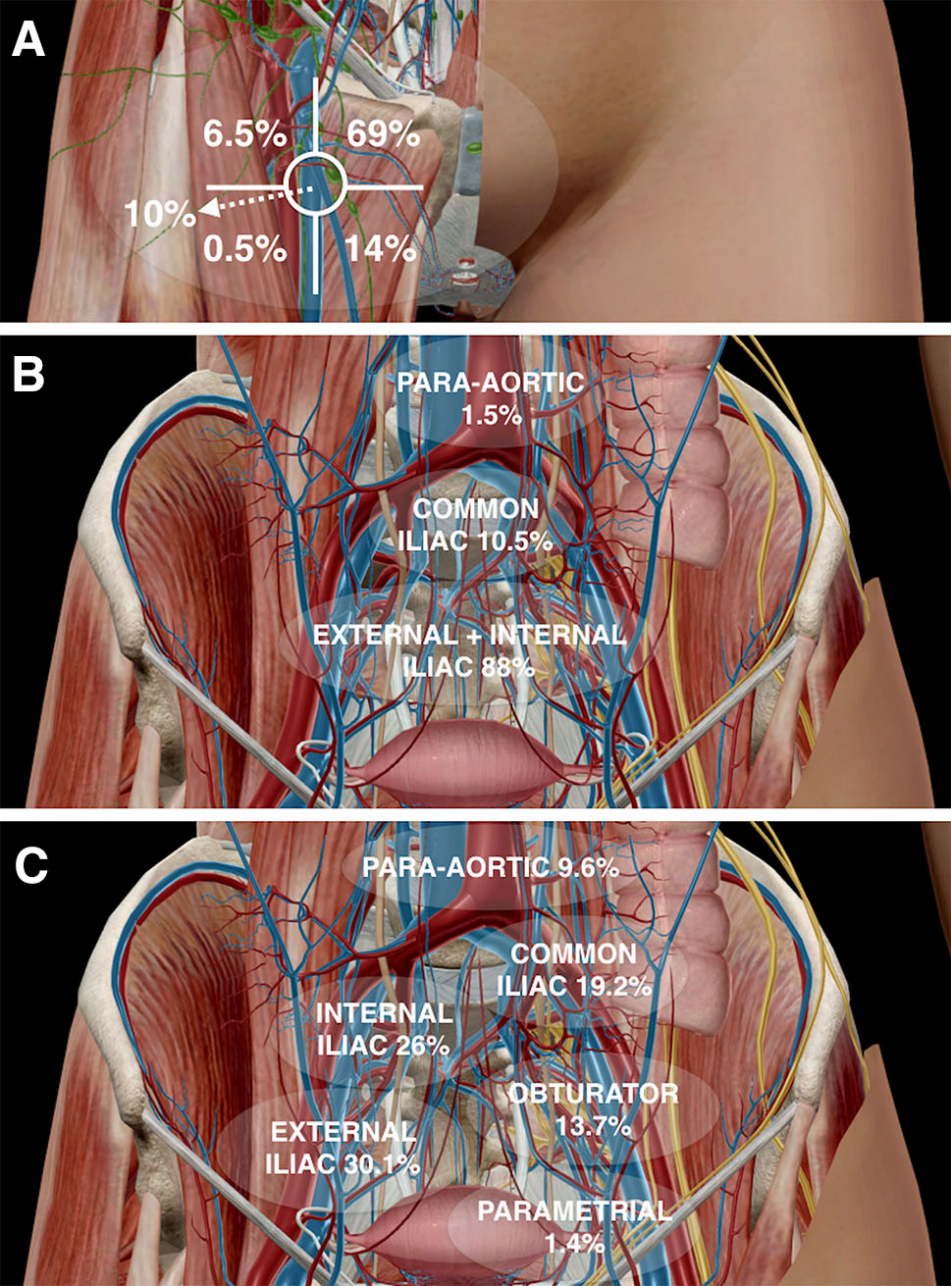
Anatomical sentinel lymph-node (SLN) distribution in gynaecological malignancies. In vulvar cancer (Ref. [48]), SLNs are limited to the groin and are predominantly found in the superior, central, and medial inferior inguinal Daseler’s zones (**a**). By contrast, in cervical cancer. (**b**) SLNs are mainly located along the iliac vessels (Ref. [15]), whereas in endometrial cancer (**c**) also para-aortic drainage is frequently observed (Ref. [17])

In gynaecological tumours, the sentinel lymph node (SLN) procedure is principally performed in vulvar cancer (VC), cervical cancer (CC), and endometrial cancer (EC). Although both preoperative lymphatic mapping and intraoperative SLN detection are common parts of SLN procedure in gynaecological tumours, the type of injection and lymphatic drainage is different for each one of these malignancies (Fig. 1). In vulvar tumour, the lymphatic drainage is predominantly superficial, and the first-draining lymph nodes are usually located in the groin. Instead, the lymphatic drainage of cervical and endometrial tumours is deep, and SLNs are located along the iliac vessels as well as in other areas with complex anatomy. Therefore, the use of preoperative SPECT/CT appears to be mandatory in cervical and endometrial tumours; whereas in vulvar tumour, it is considered more optional. In addition, intraoperative imaging, such as portable gamma-camera and intraoperative 3D navigation SPECT/CT, represents complementary tools useful to guide the surgeon in patients with difficult SLN localization, such as those close to the site of the injection or in complex anatomy areas. The new hybrid tracer using indocyanine green with ^99m^Tc-nanocolloid (ICG^99m^Tc-nanocolloid) improves the intraoperative visualization of SLN, resulting useful during the operation. All these particular aspects of SLN procedure in gynaecological malignancies will be discussed in this review. A research of the literature was performed on PubMed/MEDLINE using the following keywords (MeSH terms) to encounter the most relevant studies about the SLN procedure in gynaecology: “SLN biopsy”, “lymphatic mapping”, “lymphoscintigraphy”, “SPECT/CT”, “intraoperative SLN detection”, “hybrid tracer”, “vulvar cancer”, “cervical cancer”, and “endometrial cancer”. The search has been restricted to the English language. The references of the retrieved articles were examined to identify additional articles. This review also includes meta-analyses published in the last five years.

## Cervical cancer

### Introduction

**Fig. 2:**
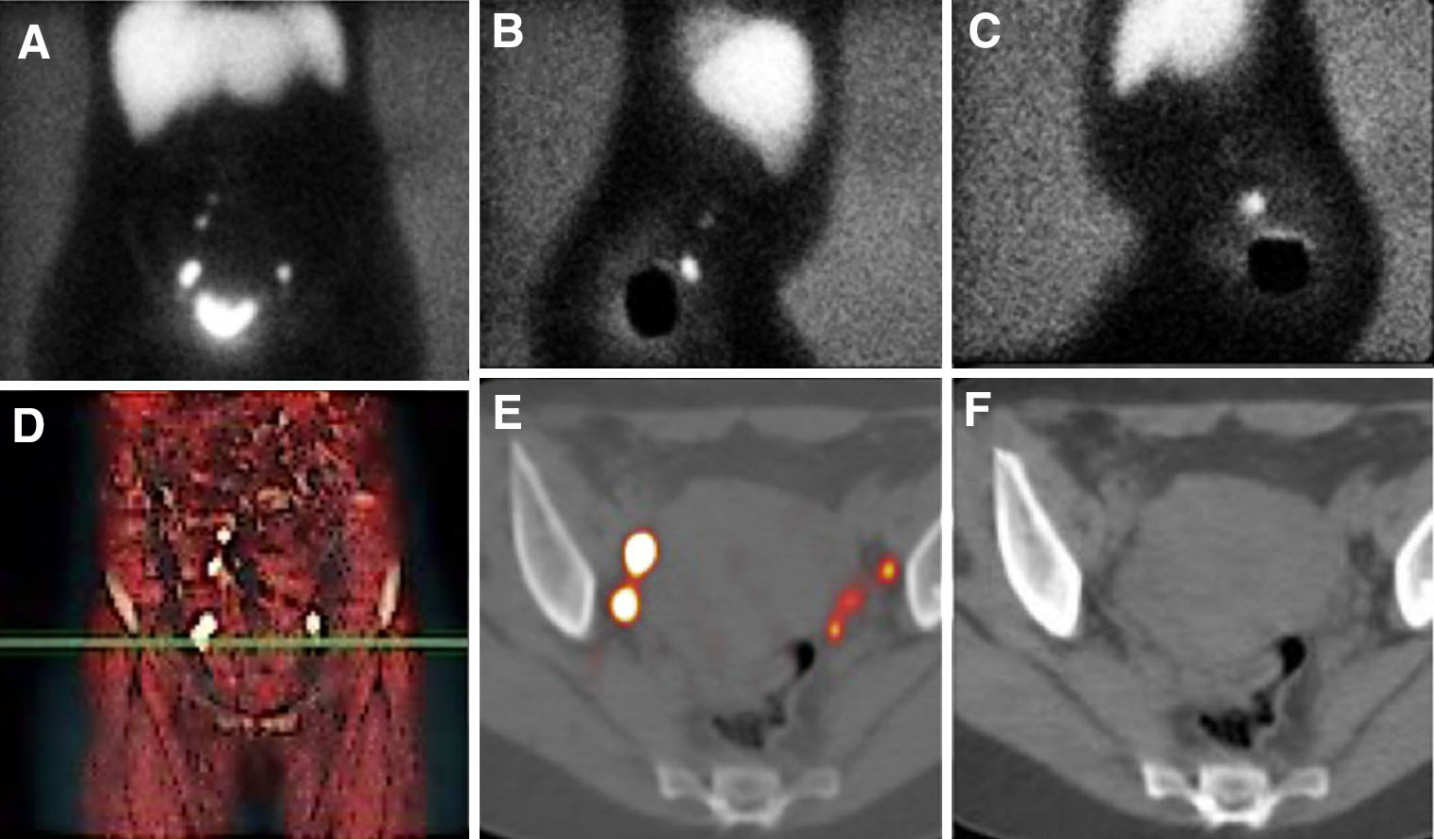
Cervical cancer. Planar images show a bilateral drainage in pelvic area (**a**–**c**). Volume-rendering image displays the level of sentinel nodes (**d**). SPECT/CT axial-fused images showing two separate nodes with high tracer uptake in right obturator fossa as well as three tiny nodes in left side (**e**). Corresponding axial CT slice (**f**)

Cervical cancer (CC) is the third most common gynaecological cancer with an estimated of 12,990 new cases and 4120 deaths in the US, in 2016 [1]. The pattern of dissemination of CC principally concerns the adjacent pelvic organs, but can also spread to locoregional lymph nodes (LN), while hematogenous spread to lung, liver, bone, and brain is rare. The most important prognostic factor is the presence of metastatic locoregional LN(s), including the pelvic- and para-aortic lymph nodes [2, 3]. According to the current guidelines, the preferred treatment for early-stage disease (FIGO stages IA-2, IB-1, IIA-1) is radical hysterectomy and SLN mapping with or without bilateral pelvic lymphadenectomy [4, 5]. The SLN(s) are the lymph node(s) that receive direct drainage from the tumour [6]; thus, the tumour status of SLN(s) reflects the status of the entire lymph node field. The SLN status plays an important role, because when an SLN contains metastases at histopathology, the best treatment approach would be based on chemo-radiotherapy. In addition, when SLNs are negative for metastases, the pelvic lymph node dissection can be safely avoided [4], reducing concomitant surgical morbidity. The uterine cervix is a midline organ; thus, lymphatic drainage is almost always bilateral and principally to the pelvic region. The most frequent localization of pelvic lymph node metastasis is the obturator followed by the external iliac basins [7]. In addition, the lymphatic drainage may spread to other areas, such as the common iliac and para-aortic basins [8]. Nevertheless, it is rare to find “skip metastasis” in the para-aortic basin without pelvic lymph node metastases [9, 10]. Therefore, the SLN mapping is useful for detection of lymphatic drainage patterns in particular to regions not routinely explored in conventional surgery, such as para-aortic chains. The SLN mapping is performed by peri-tumoural/peri-orificial injection of radiocolloid (e.g. ^99m^Tc-nanocolloid) in the four quadrants of the cervix using a 20 or 22-gauge spinal needle. In the case of previous conisation, the peri-cicatricial injection at the four quadrants is recommended [5]. The most frequently used tracer dose is approximately 110 MBq in a total volume of 2 mL [11]. The injection may be carried out the day before surgery or on the same day of surgery. The Conventional planar images are acquired for 3–5 min in anterior and lateral views at 30 (early) and 60–120 (delayed) min after injection [5]. The early images are used to visualize lymphatic duct(s) and the first-draining lymph node(s). The delayed images are used to differentiate the SLN(s) from higher echelon nodes [12]. A higher echelon node is defined as an LN draining from the SLN(s). The preoperative planar lymphoscintigraphy does not give a precise anatomical localization of the SLN(s) [13]. Therefore, SPECT in conjunction with low-dose CT (SPECT/CT) is recommended immediately after delayed imaging as a complementary modality [5], providing not just better contrast and spatial resolution in comparison to planar imaging, but also accurate anatomical information (Fig. 2).

### Advantages of preoperative SPECT/CT imaging

SPECT/CT has higher SLN detection rate compared to the conventional planar images (98.6 vs. 85.3 %), as reported in a recent meta-analysis, including eight studies [14]. In general, SPECT/CT provides an accurate anatomical SLN localization [15–18]. In particular, SPECT/CT images are useful to detect SLN(s) close to injection sites, such as parametrial SLNs, as well as SLN(s) in uncommon locations, such as the para-aortic and presacral basins [15, 17, 19]. In addition, SPECT/CT leads to better detection of bilateral SLN(s) compared to planar imaging [20–22] (Table 1). Furthermore, Hoogendam et al. reported that SPECT/CT provides a valuable surgical roadmap, reducing the surgical time in cervical cancer in women undergoing robotic-assisted surgery [22]. Recently, ^99m^Tc SPECT/MRI-fused images have been used for SLN mapping in preoperative assessment of SLN metastases in the early-stage cervical cancer in women. The authors found a ^99m^Tc SPECT-MRI accuracy of 74.9 % (95 % CI 0.569–0.930) to non-invasively assess SLN metastases, including 136 SLNs of which 13 (9.6 %) in 8/79 patients (10.7 %) contained metastases [23].

**Table 1:** Detection of sentinel nodes in cervical cancer using planar lymphoscintigraphy and SPECT/CT

Authors	Year	Study type	*N*	Radiotracer (dosing)	Detection rate by LSG (%)	Detection rate by SPECT/CT (%)	SLNs detected by LSG	SLNs detected by SPECT/CT	Bilateral SLN detected by LSG (%)	Bilateral SLN detected by SPECT/CT (%)	False negative rate (%)
Martinez [15]	2010	Retrospective	41	^99m^Tc- sulfur rhenium colloid (80 MBq)	N/A	95	N/A	86	N/A	49	0
Pandit [17]	2010	Prospective	10	^99m^Tc-sulfur colloid (37-148 MBq)	70	100	26	51	N/A	N/A	0
Diaz [18]	2011	Prospective	22	^99m^Tc-albumin nanocolloid (144 MBq)	100	100	35	40	N/A	N/A	0
Kraft [16]	2012	Retrospective	36	^99m^Tc-nanocolloid (40 MBq)	89	97	N/A	N/A	N/A	N/A	N/A
Buda [39]	2012	Retrospective	10	^99m^Tc-albumin nanocolloid (30-40 MBq)	80	100	N/A	N/A	N/A	N/A	0
Belhoncine [19]	2013	Prospective	7	^99m^Tc-cysteine rhenium colloid (37 MBq)	86	100	15	23	N/A	N/A	0
Bournaud [21]	2013	Retrospective	42^a^	^99m^Tc-sulfur rhenium colloid (60-120 MBq)	95	95	152	173	70	73	0
Hoogendam [22]	2013	Retrospective	62^b^	^99m^Tc-nanocolloid (220-290 MBq)	85	93	71	58	76	79	5
Klapdor [20]	2014	Prospective	51	^99m^Tc-nanocolloid (10 MBq)	84	92	N/A	N/A	57	64	0

*N* number of patients, *LSG* lymphoscintigraphy, *SLNs* sentinel lymph nodes, *N/A* not available

^a^No lymphoscintigraphy was performed in 3 of 42 patients

^b^33 pts underwent LSG and 29 pts underwent SPECT/CT

## Endometrial cancer

### Introduction

Endometrial cancer (EC) is the most common malignancy of gynaecological cancer with an estimated incidence of 60,050 new cases and 10,470 deaths in the US, in 2016 [1]. Lymph-node status is a key prognostic factor in endometrial tumours. Indeed, the 5-year survival rate varies from 44 to 52 % when pelvic- or para-aortic node lymph nodes contained metastases [24]. Radical pelvic- and para-aortic lymphadenectomies represent the standard treatment in high-risk group (grade 3, >50 % myometrial invasion) or high-risk tumour histology (papillary serous, carcinosarcoma, and clear cell cancer) [25]. The SLN technique may provide the surgical staging, avoiding the morbidity of complete lymphadenectomy in patients with negative SLN biopsy, but also ultra-staging assessment (micro-metastases and isolated tumour cells) through extensive immunochemistry. Although there are several studies validating SLN mapping in EC, this technique is not yet the standard of care in the early-stage EC (Stage I-II high-risk) [5, 25]. One of the most controversial aspects for SLN mapping is the modality of injection. Indeed, three different modalities of injection have been described in the currently literature: (i) cervical injection; (ii) endometrial peri-tumoural injection assisted by hysteroscopy; and (iii) myometrial/subserosal intraoperative injection. The radiotracer can be injected on the day prior to surgery, providing lymphatic mapping with planar and SPECT/CT images. The most common and easiest approach is the cervical injection, which is performed peri-orificially into the four quadrants as well as for CC. The detection rate related to cervical injection is the highest of the three injection modalities used in endometrial cancer, ranging from 62 to 100 % [26]. Endometrial radiotracer administration assisted by hysteroscopy allows direct injection around the tumour. This procedure is usually performed at the beginning of the surgery, without the possibility to obtain preoperative SLN mapping. The detection rate of this injection modality varies from 40 to 95 % [27–31]. Finally, myometrial/subserosal injection is performed during the surgery and has been predominantly limited to the use of blue dye administered at a minimum of three locations [32]. This injection route is associated with a detection rate varying from 45 to 91 % [32–35]. Robova et al. compared subserosal injection (using blue dye and radiotracer) with hysteroscopic injection (radiotracer only) in 67 and 24 patients, respectively; although the detection rate was 73 % with subserosal injection and 50 % with hysteroscopic injection [36]; the authors concluded that both injection routes provide insufficient SLN identification. An alternative modality for radiotracer administration has been recently introduced using myometrial/subserosal injection guided by transvaginal ultrasonography; with this technique, a high detection rate (88 %) can be reached when a high-injected volume (8 mL) is achieved [37].

### Additional value of preoperative SPECT/CT imaging

**Fig. 3:**
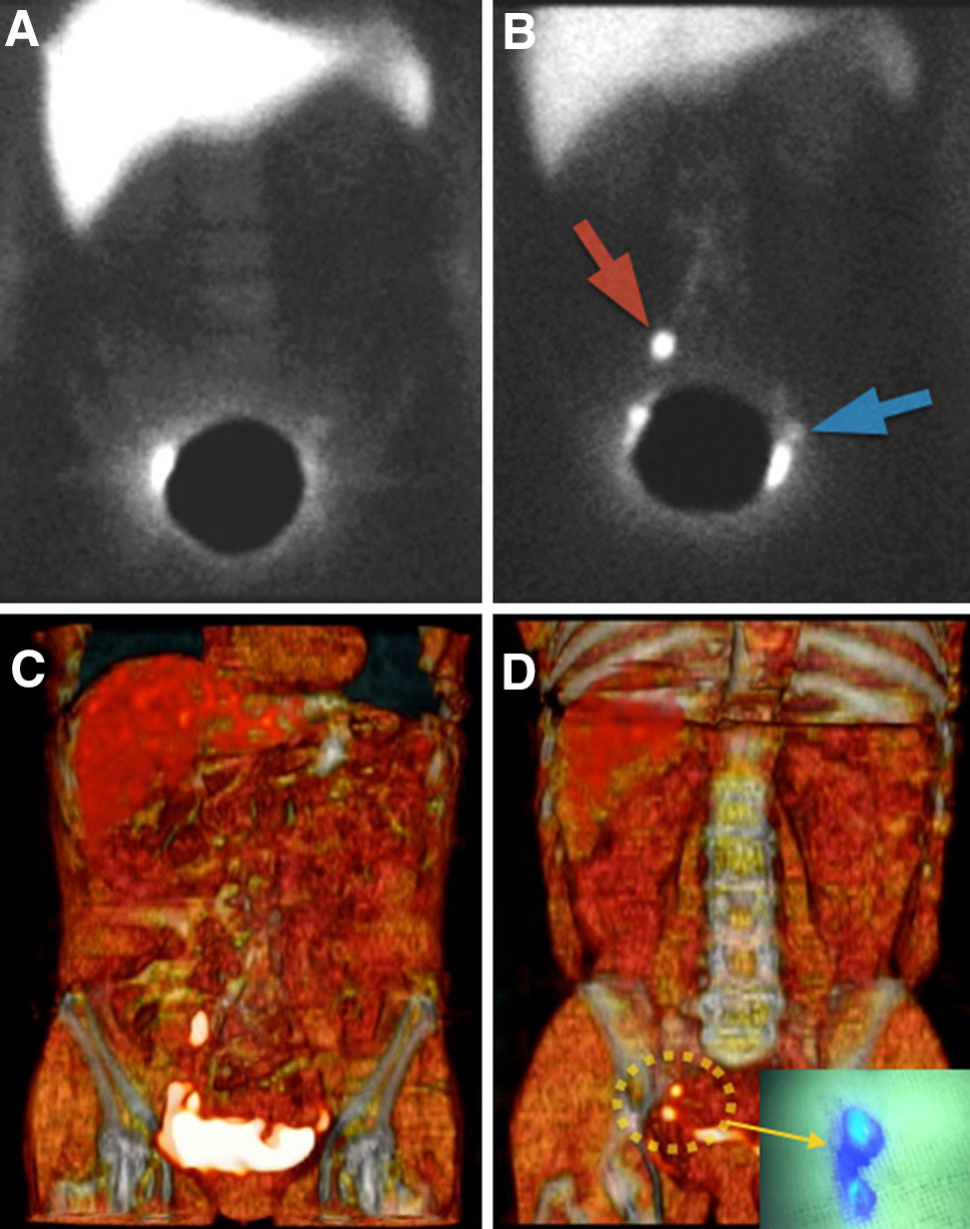
Endometrial cancer. Early planar image showed a very faint left node. A lied shield covered the injection area and high activity on the right side was supposed to be a partial zone of the injection area (**a**). Delayed planar image displays a right sentinel node (*red arrow*) and the previously observed left sentinel node (*blue arrow*). 3D volume-rendering image shows the same node distribution like **b** (**c**). A more detailed analysis of SPECT/CT data and 3D reconstructed images showed two posterior and caudal nodes (*dotted circle*) previous to the marked as sentinel node in **b** corresponding to external iliac nodes during surgery (*arrow*)

The deep lymphatic drainage of the corpus uteri is a probable reason for the low correlation found between planar lymphoscintigraphy and surgical mapping [38]. This limiting factor may be solved when SPECT/CT is performed in addition to planar images (Fig. 3). This fused SPECT/CT is useful in areas of deep lymphatic drainage, such as the pelvis, providing correction for tissue attenuation with detection of additional SLN(s) in other basins accompanied by accurate anatomical localization. Therefore, preoperative SPECT/CT plays an important role in the planning of surgery and may lead to a decrease of surgical time. Until now, there are few articles reporting the use of SPECT/CT in endometrial cancer [16, 17, 39]. Pandit-Taskar et al. have reported a series, including 40 patients, with endometrial tumour; the authors showed a higher detection rate using SPECT/CT (100 %) compared to a planar lymphoscintigraphy (75 %), a hand-held probe (93 %), and blue dye alone (83 %), and highlighted the ability of SPECT/CT to detect additional SLN(s) in the para-aortic basin [17]. More recently, Naaman et al. reported in 53 endometrial cancer patients that SPECT/CT contributed to increase SLN visualization from 67 %, when only planar lymphoscintigraphy was used, to 84 % when SPECT/CT was included; in this series, anatomical accuracy of SPECT/CT was 91 % [40] (Table 2).

**Table 2:** Detection of sentinel nodes in endometrial cancer using planar lymphoscintigraphy and in SPECT/CT

Authors	Year	Study type	*N*	Injection site	Radiotracer (dosing)	Detection rate by LSG (%)	Detection rate by SPECT/CT (%)	SLNs detected by LSG	SLNs detected by SPECT/CT	Bilateral SLN detection by LSG (%)	Bilateral SLN detection by SPECT/CT (%)	False negative rate (%)
Pandit [17]	2010	Prospective	40	C	^99m^Tc-sulfur colloid (37-148 MBq)	75	100	67	207	N/A	N/A	0
Kraft [16]	2012	Retrospective	21	C	^99m^Tc-nanocolloid (40 MBq)	86	86	N/A	N/A	N/A	N/A	N/A
Buda [39]	2012	Prospective	25	C	^99m^Tc-albumin nanocolloid (30-40 MBq)	40	88	N/A	N/A	N/A	N/A	0
Naaman [40]	2016	Retrospective	45^a^	C	^99m^Tc-albumin nanocolloid (7.4-1.1 MBq)	67	84	N/A	N/A	29	49	0

*N* number of patients, *C* cervix, *LSG* lymphoscintigraphy, *SLNs* sentinel lymph nodes, *N/A* not available

^a^Only 37 of 45 underwent SPECT/CT

## Vulvar cancer

### Introduction

Vulvar cancer (VC) is a rare gynaecological malignancy with an estimated number of 5950 new cases and 1110 deaths in the US, in 2016 [1]. The pattern of dissemination is principally lymphogenic, with drainage first to the superficial inguinal nodes, then to the deep inguinal nodes and, finally, to the pelvic lymph nodes. Therefore, the presence of metastatic lymph node represents the most important prognostic factor. Indeed, the 5-year survival rate decreases from 94.7 %, when the LNs are negative, to 62 % when containing metastases [41]. The current standard treatment includes radical vulvectomy with SLN procedure and/or inguinofemoral lymphadenectomy. In particular, the sentinel lymph node biopsy is recommended in the early squamous cell vulvar carcinoma (Stage FIGO 2009: Ib/II) with unifocal tumours less than 4 cm in size and clinically negative (cN0) lymph nodes in the groins [5, 42, 43].

The SLN mapping is performed through injection of radiocolloid (e.g. ^99m^Tc-nanocolloid) in three or four intradermal/intramucous around the primary lesion or excision scar a few minutes after the application of an anaesthetic lidocaine spray or crème. Simultaneous anterior and lateral dynamic lymphoscintigraphy is performed immediately after injection followed by early (15 min) and delayed (2 h) static planar imaging. Subsequently, SPECT/CT is recommended as complementary modality, providing anatomical and functional information facilitating more accurate virtual surgical planning [5]. The SLN detection rate using radiotracer injection has been found to be higher than 95 % [44, 45]. In a recent meta-analysis, the SLN detection rate per groin using radiocolloid and blue dye was 87 % (range 82–92 %), the false negative rate 6.4 % (range 4.4–8.8 %), and the recurrence rate 2.8 % (range 1.5–4.4 %) [42]

### Additional value of preoperative SPECT/CT imaging

**Fig. 4:**
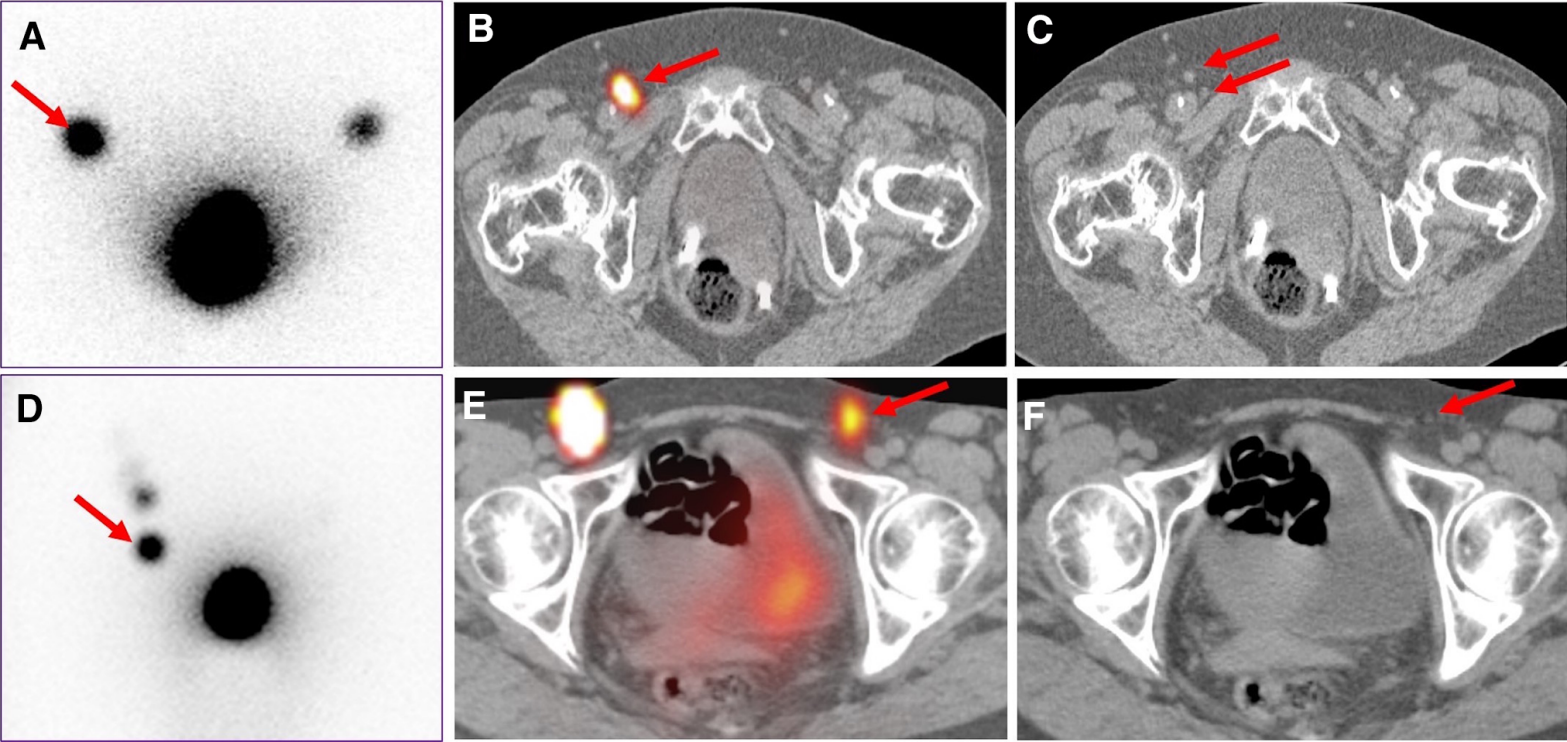
In a patient with vulvar cancer, delayed planar imaging (**a**) shows one SLN in the right groin (*red arrow*) corresponding with one allocated SLN uptake (*red arrow*) on transversal-fused SPECT/CT (**b**) and two not enlarged lymph nodes on transversal CT (**c**) (*double arrows*). In another patient, delayed planar image (**d**) shows unilateral lymphatic drainage with a single SLN in the right groin (*red arrow*), while transversal-fused SPECT/CT (**e**) shows bilateral drainage with also a contralateral SLN (*red arrow*) corresponding with a not enlarged lymph node in the left groin on CT (**f**)

As reported in the current literature, SPECT/CT plays an important role to provide a better anatomical localization of SLN(s) and to detect additional lymph nodes in the same region or in other regions with poor or even without visualization at planar lymphoscintigraphy (Fig. 4), as well as to reduce the false positive rate possibly due to external contamination or presence of radioactivity in enlarged lymphatic vessels, [16, 19, 46, 47]. Recently, Collarino et al. reported the use of SPECT/CT for anatomical mapping of lymphatic drainage in vulvar cancer. According to the five Daseler zones using the inguinal saphenofemoral junction as anatomical reference, the authors found that the lymphatic drainage was principally to the medial inguinal region (83 %), and the drainage to the lateral inferior groin was only incidental (0.5 %) in 83 patients with cN0 vulvar cancer (Fig. 1). Further drainage to higher echelon nodes was visualized in the groin (15 %) and in the pelvis (85 %). Therefore, SPECT/CT is able to personalize the lymphatic mapping, and has a potential role in limiting the extent of lymph node dissection to the lateral inferior zone in patients with positive SLN(s) [48] (Table 3).

**Table 3:** Detection of sentinel nodes in vulvar cancer using planar lymphoscintigraphy and SPECT/CT

Authors	Year	Study type	*N*	Radiotracer (dosing)	Detection rate by LSG (%)	Detection rate by SPECT/CT (%)	SLNs detected by LSG	SLNs detected by SPECT/CT	False negative rate (%)
Beneder [46]	2008	Prospective	10	^99m^Tc-nanocolloid (60 MBq)	N/A	N/A	26	38	0
Kraft [16]	2012	Retrospective	7	^99m^Tc-nanocolloid (40 MBq)	100	100	N/A	N/A	N/A
Belhoncine [19]	2013	Prospective	7	^99m^Tc-cysteine rhenium colloid (37 MBq)	86	100	N/A	N/A	0
Mathéron [49]	2013	Prospective	14	ICG-^99m^Tc-nanocolloid (87 MBq)	N/A	N/A	39	39	0
Collarino [48]	2015	Retrospective	83	^99m^Tc-nanocolloid (81 MBq)	N/A	N/A	192	217	0

*N* number of patients, *LSG* lymphoscintigraphy, *SLNs* sentinel lymph nodes, *N/A* not available, *ICG* indocyanine green

## Advancements in intraoperative imaging and instrumentation

The indocyanine green (ICG) added to ^99m^Tc-nanocolloid in one signature represents a new hybrid tracer for the detection of SLN. Recently, Mathéron et al. reported the use of a new hybrid tracer, in the SLN identification procedure in vulvar cancer. They showed that 98 % of the SNs were radioactive at the time of excision, 96 % were fluorescent, and only 65 % were blue in 15 patients. The additional value of ICG is related to better intraoperative visualization of SN to optimize the intraoperative SLN visualization using the fluorescence component [49]. Nevertheless, there are no studies available on application of this hybrid tracer in cervical cancer and endometrial cancer. Indeed, a recently meta-analysis, including 67 studies in cervical cancer reported only the SLN detection rate using the combination of radiotracer with blue dye compared to the single use of radiotracer, blue dye, and florescence imaging (92.3 vs. 90.9 vs. 80.9 vs 76.5 %) [50]. In the future, the ICG-^99m^Tc-nanocolloid may be used in the SLN(s) identification procedure during the robot-assisted laparoscopy [51, 52]. In addition, a portable γ-camera might be a complementary tool during surgery in gynaecological cancer. With a portable γ-camera an intraoperative real-time imaging is acquired before SLN resection. Subsequently, an additional image of the surgical field is performed to confirm the absence of any residual activity after excision. This device may improve the intraoperative detection rate in patients with difficult SLN localization in parametrial and precaval lymph-node basins. Indeed, the hand-held γ-probe has limitations to detect parametrial SLN(s) due to their location in the vicinity of the injection site, and in precaval SLN(s) because of the liver activity. Vidal-Sicart et al. reported the use of portable γ-camera in gynaecological cancer, showing a higher detection (92 %) when compared to just hand-held γ-probe (77 %) in the two cases of high-risk endometrial cancer, three cases of cervical cancer and one patients with vulvar cancer [53]. Furthermore, the incorporation of co-registered SPECT/CT to 3D navigation probe may be used during the operation offering a 3D roadmap to the surgeon and facilitating the anatomical localization of SLN(s). In the current literature on radioguided surgery, this approach has been reported in penile cancer by Brower et al. [54].

## Conclusion

In conclusion, the SLN procedure has widely been validated in vulvar cancer and cervical cancer. Its application in these malignancies is well standardized and has been incorporated to the current guidelines in Europe and North America. By contrast, in endometrial cancer, there are various controversial aspects (e.g. injection route) to be clarified and the use of the SLN procedure needs to be validated in larger clinical series. Beside these aspects, the present review showed that recent technological advances, such as preoperative and intraoperative use of SPECT/CT, the contribution of the hybrid tracer ICG-99mTc-nanocolloid, and technological advances like SLN robotic-guided procedure might play an increasing role to guide gynaecological cancer surgery in the future.

## References

[1] Siegel RL, Miller KD, Jemal S (2016). Cancer statistics. CA Cancer J Clin.

[2] Stehman FB, Bundy BN, DiSaia PJ, Keys HM, Larson JE, Fowler WC (1991). Carcinoma of the cervix treated with radiation therapy. I. A multi-variate analysis of prognostic variables in the Gynecologic Oncology Group. Cancer.

[3] Macdonald OK, Chen J, Dodson M, Lee CM, Gaffney DK (2009). Prognostic significance of histology and positive lymph node involvement following radical hysterectomy in carcinoma of the cervix. Am J Clin Oncol.

[4] Koh WJ, Greer BE, Abu-Rustum NR, Apte SM, Campos SM, Cho KR (2015). Cervical cancer, version 2.2015. J Natl Compr Canc Netw.

[5] Giammarile F, Bozkurt MF, Cibula D, Pahisa J, Oyen WJ, Paredes P, Olmos RV, Sicart SV (2014). The EANM clinical and technical guidelines for lymphoscintigraphy and sentinel node localization ingynaecological cancers. Eur J Nucl Med Mol Imaging.

[6] Nieweg OE (2012) The sentinel lymph node concept in oncology surgery. In: Mariani G, Manca G, Orsini P, Vidal-Sicar t S, Valdés Olmos R (eds) Atlas of lymphoscintigraphy and sentinel node mapping. Springer, Milan, pp 87–93

[7] Benedetti-Panici P, Maneschi F, Scambia G, Greggi S, Cutillo G, D’Andrea G, Rabitti C, Coronetta F, Capelli A, Mancuso S (1996). Lymphatic spread of cervical cancer: an anatomical and pathological study based on 225 radical hysterectomies with systematic pelvic and aortic lymphadenectomy. Gynecol Oncol.

[8] Sakuragi N, Satoh C, Takeda N, Hareyama H, Takeda M, Yamamoto R, Fujimoto T, Oikawa M, Fujino T, Fujimoto S (1999). Incidence and distribution pattern of pelvic and paraaortic lymph node metastasis in patients with stages IB, IIA, and IIB cervical carcinoma treated with radical hysterectomy. Cancer.

[9] Lea JS, Sheets EE, Duska LR, Miller DS, Schorge JO (2002). Early-stage cervical adenocarcinoma treated by surgical intent: the role of paraaortic lymph node dissection. Gynecol Oncol.

[10] Bader AA, Winter R, Haas J, Tamussino KF (2007) Where to look for the sentinel lymph node in cervical cancer. Am J Obstet Gynecol 197(6):678.e1–7 10.1016/j.ajog.2007.09.053 18060980

[11] El-Ghobashy AE, Saidi SA (2009). Sentinel lymph node sampling in gynaecological cancers: techniques and clinical applications. Eur J Surg Oncol.

[12] Paredes P, Vidal-Sicart S (2012) Preoperative and intraoperative lymphatic mapping for radioguided sentinel node biopsy in cancers of the female reproductive system. In: Mariani G, Manca G, Orsini P, Vidal-Sicar t S, Valdés Olmos R (eds). Atlas of lymphoscintigraphy and sentinel node mapping. Springer, Milan, pp 249–268

[13] Vermeeren L, van der Ploeg IM, Olmos RA, Meinhardt W, Klop WM, Kroon BB, Nieweg OE (2010). SPECT/CT for preoperative sentinel node localization. J Surg Oncol.

[14] Hoogendam JP, Veldhuis WB, Hobbelink MG, Verheijen RH, van den Bosch MA, Zweemer RP (2015). 99 mTc SPECT/CT versus planar lymphoscintigraphy for preoperative sentinel lymph node detection in cervical cancer: a systematic review and metaanalysis. J Nucl Med.

[15] Martínez A, Zerdoud S, Mery E, Bouissou E, Ferron G, Querleu D (2010). Hybrid imaging by SPECT/CT for sentinel lymph node detection in patients with cancer of the uterine cervix. Gynecol Oncol.

[16] Kraft O, Havel MD (2012). Detection of sentinel lymph nodes in gynecologic tumours by planar scintigraphy and SPECT/CT. Mol Imaging Radionucl Ther.

[17] Pandit-Taskar N, Gemignani ML, Lyall A, Larson SM, Barakat RR, Abu Rustum NR (2010). Single photon emission computed tomography SPECT-CT improves sentinel node detection and localization in cervical and uterine malignancy. Gynecol Oncol.

[18] Díaz-Feijoo B, Pérez-Benavente MA, Cabrera-Diaz S, Gil-Moreno A, Roca I, Franco-Camps S, Fernández MS, García-Jiménez A, Xercavins J, Martínez-Palones JM (2011). Change in clinical management of sentinel lymph node location in early stage cervical cancer: the role of SPECT/CT. Gynecol Oncol.

[19] Belhocine TZ, Prefontaine M, Lanvin D, Bertrand M, Rachinsky I, Ettler H, Zabel P, Stitt LW, Sugimoto A, Urbain JL (2013). Added-value of SPECT/CT to lymphatic mapping and sentinel lymphadenectomy in gynaecological cancers. Am J Nucl Med Mol Imaging.

[20] Klapdor R, Mucke J, Schneider M, Länger F, Gratz KF, Hillemanns P, Hertel H (2014). Value and advantages of preoperative sentinel lymph node imaging with SPECT/CT in cervical cancer. Int J Gynecol Cancer.

[21] Bournaud C, Le Bail-Carval K, Scheiber C, de Charry C, Mathevet P, Moreau-Triby C (2013). Value of SPECT/CT in lymphatic mapping in cervix and endometrial cancer. Méd Nucl.

[22] Hoogendam JP, Hobbelink MG, Veldhuis WB, Verheijen RH, van Diest PJ, Zweemer RP (2013). Preoperative sentinel node mapping with (99 m)Tc-nanocolloid SPECT-CT significantly reduces the intraoperative sentinel node retrieval time in robot assisted laparoscopic cervical cancer surgery. Gynecol Oncol.

[23] Hoogendam JP, Zweemer RP, Hobbelink MG, van den Bosch MA, Verheijen RH, Veldhuis WB (2016). 99mTc-nanocolloid SPECT-MRI fusion for the selective assessment of non-enlarged sentinel lymph nodes in patients with early stage cervical cancer. J Nucl Med.

[24] Partridge EE, Shingleton HM, Menck HR (1996). The national cancer data base report on endometrial cancer. J Surg Oncol.

[25] National Comprehensive Cancer Network, (2016) Uterine neoplasm, version 2.2016. http://www.nccn.org/professionals/physician_gls/pdf/uterine.pdf. Accessed 28 March 2016

[26] Cormier B, Rozenholc AT, Gotlieb W, Plante M, Giede C, Communities of Practice (CoP) Group of Society of Society of Gynecologic Oncology of Canada (GOC) (2015). Sentinel lymph node procedure in endometrial cancer: a systematic review and proposal for standardization offuture research. Gynecol Oncol.

[27] Gien LT, Kwon JS, Carey MS (2005). Sentinel node mapping with isosulfan blue dye in endometrial cancer. J Obstet Gynaecol Can.

[28] Maccauro M, Lucignani G, Aliberti G, Villano C, Castellani MR, Solima E, Bombardieri E (2005). Sentinel lymph node detection following the hysteroscopic peritumoural injection of 99mTc-labelled albumin nanocolloid in endometrial cancer. Eur J Nucl Med Mol Imaging.

[29] Delaloye JF, Pampallona S, Chardonnens E, Fiche M, Lehr HA, DeGrandi P, Delaloye AB (2007). Intraoperative lymphatic mapping and sentinel node biopsy using hysteroscopy in patients with endometrial cancer. Gynecol Oncol.

[30] Clement D, Bats AS, Ghazzar-Pierquet N, Le Frere Belda MA, Larousserie F, Nos C, Lecuru F (2008). Sentinel lymph nodes in endometrial cancer: is hysteroscopic injection valid?. Eur J Gynaecol Oncol.

[31] Solima E, Martinelli F, Ditto A, Maccauro M, Carcangiu M, Mariani L, Kusamura S, Fontanelli R, Grijuela B, Raspagliesi F (2012). Diagnostic accuracy of sentinel node in endometrial cancer by using hysteroscopic injection of radiolabeled tracer. Gynecol Oncol.

[32] Frumovitz M, Bodurka DC, Broaddus RR, Coleman RL, Sood AK, Gershenson DM, Burke TW, Levenback CF (2007). Lymphatic mapping and sentinel node biopsy in women with high-risk endometrial cancer. Gynecol Oncol.

[33] Altgassen C, Pagenstecher J, HornungD Diedrich K, Hornemann A (2007). A new approach to label sentinel nodes in endometrial cancer. Gynecol Oncol.

[34] Li B, Li XG, Wu LY, Zhang WH, Li SM, Min C, Gao JZ (2007). A pilot study of sentinel lymph nodes identification in patients with endometrial cancer. Bull Cancer.

[35] Lopes LA, Nicolau SM, Baracat FF, Gonçalves WJ, Santos HV, Lopes RG, Lippi UG (2007). Sentinel lymph node in endometrial cancer. Int J Gynecol Cancer.

[36] Robova H, Charvat M, Strnad P, Hrehorcak M, Taborska K, Skapa P, Rob L (2009). Lymphatic mapping in endometrial cancer: comparison of hysteroscopic and subserosal injection and the distribution of sentinel lymph nodes. Int J Gynecol Cancer.

[37] Torné A, Pahisa J, Vidal-Sicart S, Martínez-Roman S, Paredes P, Puerto B, Albela S, Fusté P, Perisinotti A, Ordi J (2013). Transvaginal ultrasound-guided myometrial injection of radiotracer (TUMIR): a new method for sentinel lymph node detection in endometrial cancer. Gynecol Oncol.

[38] Ballester M, Rouzier R, Countant C, Kerrou K, Daraï E (2009). Limits of lymphoscintigraphy for sentinel node biopsy in women with endometrial cancer. Gynecol Oncol.

[39] Buda A, Elisei F, Arosio M, Dolci C, Signorelli M, Perego P, Giuliani D, Recalcati D, Cattoretti G, Milani R, Messa C (2012). Integration of hybrid single-photon emission computed tomography/computed tomography in the preoperative assessment of sentinel node in patients with cervical and endometrial cancer: our experience and literature review. Int J Gynecol Cancer.

[40] Naaman Y, Pinkas L, Roitman S, Ikher S, Oustinov N, Vaisbuch E, Yachnin A, Ben-Arie A (2016). The added value of SPECT/CT in sentinel lymph nodes mapping for endometrial carcinoma. Ann Surg Oncol.

[41] Burger MP, Hollema H, Emanuels AG, Krans M, Pras E, Bouma J (1995). The importance of the groin node status for the survival of T1 and T2 vulval carcinoma patients. Gynecol Oncol.

[42] Covens A, Vella ET, Kennedy EB, Reade CJ, Jimenez W, Le T (2015). Sentinel lymph node biopsy in vulvar cancer: systematic review, meta-analysis and guideline recommendations. Gynecol Oncol.

[43] National Comprehensive Cancer Network (2016) Vulvar cancer (squamous cell carcinoma), version .2016 http://www.nccn.org/professionals/physician_gls/pdf/vulvar.pdf. Accessed 28 March 2016

[44] Hampl M, Hantschmann P, Michels W, Hillemanns P, German Multicenter Study Group (2008). Validation of the accuracy of the sentinel lymph node procedure in patients with vulvar cancer: results of a multicenter study in Germany. Gynecol Oncol.

[45] Vidal-Sicart S, Puig-Tintoré LM, Lejárcegui JA, Paredes P, Ortega ML, Muñoz A, Ordi J, Fusté P, Ortín J, Duch J, Martín F, Pons F (2007). Validation and application of the sentinel lymph node concept in malignant vulvar tumours. Eur J Nucl Med Mol Imaging.

[46] Beneder C, Fuechsel FG, Krause T, Kuhn A, Mueller MD (2008). The role of 3D fusion imaging in sentinel lymphadenectomy for vulvar cancer. Gynecol Oncol.

[47] Valdés Olmos RA, Rietbergen DD, Vidal-Sicart S, Manca G, Giammarile F, Mariani G (2014). Contribution of SPECT/CT imaging to radioguided sentinel lymph node biopsy in breast cancer, melanoma, and other solid cancers: from “open and see” to “see and open”. Q J Nucl Med Mol Imaging.

[48] Collarino A, Donswijk ML, van Driel WJ, Stokkel MP, Valdés Olmos RA (2015). The use of SPECT/CT for anatomical mapping of lymphatic drainage in vulvar cancer: possible implications for the extent of inguinal lymph node dissection. Eur J Nucl Med Mol Imaging.

[49] Mathéron HM, van den Berg NS, Brouwer OR, Kleinjan GH, van DrielWJ TrumJW, Vegt E, Kenter G, van Leeuwen FW, Valdés Olmos RA (2013). Multimodal surgical guidance towards the sentinel node in vulvar cancer. Gynecol Oncol.

[50] Kadkhodayan S, Hasanzadeh M, Treglia G, Azad A, Yousefi Z, Zarifmahmoudi L, Sadeghi R (2015). Sentinel node biopsy for lymph node staging of uterine cervix cancer: systematic review and meta-analysis of the pertinent literature. Eur J Surg Oncol.

[51] KleinJan GH, van den Berg NS, Brouwer OR, de Jong J, Acar C, Wit EM, Vegt E, van der Noort V, Valdés Olmos RA, van Leeuwen FW, van der Poel HG (2014). Optimisation of fluorescence guidance during robot-assisted laparoscopic sentinel node biopsy for prostate cancer. Eur Urol.

[52] KleinJan GH, van den Berg NS, de Jong J, Wit EM, Thygessen H, Vegt E, van der Poel HG, van Leeuwen FW (2016) Multimodal hybrid imaging agents for sentinel node mapping as a means to (re)connect nuclear medicine to advances made in robot-assisted surgery. Eur J Nucl Med Mol Imaging 43(7):1278–87. doi:10.1007/s00259-015-3292-2 10.1007/s00259-015-3292-2 PMC4865539 26768422

[53] Vidal-Sicart S, Paredes P, Zanón G, Pahisa J, Martinez-Román S, Caparrós X, Vilalta A, Rull R, Pons F (2010). Added value of intraoperative real-time imaging in searches for difficult-to-locate sentinel nodes. J Nucl Med.

[54] Brouwer OR, van den Berg NS, Mathéron HM, Wendler T, van der Poel HG, Horenblas S, Valdés Olmos RA, van Leeuwen FW (2014). Feasibility of intraoperative navigation to the sentinel node in the groin using preoperatively acquired single photon emission computerized tomography data: transferring functional imaging to the operating room. J Urol.

